# Development and Optimization of Lipid-polymer Hybrid Nanoparticles Containing Melphalan Using Central Composite Design and Its Effect on Ovarian Cancer Cell Lines

**DOI:** 10.22037/ijpr.2021.114575.14923

**Published:** 2021

**Authors:** Seyedeh Masoomeh Sadat Mirnezami, Amir Heydarinasab, Azim Akbarzadehkhyavi, Mehdi Adrjmand

**Affiliations:** a *Department of Chemical Engineering, Science and Research Branch, Islamic Azad University, Tehran, Iran* *.*; b *Department of Pilot Nano-biotechnology, Pasteur Institute of Iran, Tehran, Iran. *; c *Department of Chemical Engineering, South Tehran Branch, Islamic Azad University, Tehran, Iran.*

**Keywords:** HLPNPs, Melphalan, Central Composite Design, Nanoprecipitation, MTT assay, ovarian cancer

## Abstract

The development of controlled-release drug delivery systems has a great potential to improve the efficacy of anticancer drugs. This study aimed to develop and optimize the production of hybrid lipid-polymer nanoparticles (*HLPNPs*) for the targeted delivery of *melphalan* anticancer drugs. Response surface methodology (*RSM*) and central composite design (*CCD*) were used to evaluate and optimize the effects of three independent variables including lipid, polymer, and polyvinyl alcohol (*PVA*) ratios on the nanoparticles (*NPs*) size and drug entrapment efficiency (*EE*%). Hybrid NPs were prepared using the nanoprecipitation method. The results demonstrated that spherical *NPs* were synthesized, and the rate of *EE%* went up by increasing the polymer as well as decreasing the *PVA* concentrations. The nanoformulation released *melphalan* in a sustained and controlled manner (17.39% in a period time of 48 h). Also, cytotoxicity evaluations showed that *HLPNPs *caused an increase in the efficacy of *melphalan* against human ovarian *A2780CP* and *SKOV3* cancer cells. Overall, the results of this study demonstrated that *HLPNPs* can be considered as a promising carrier for the delivery of hydrophobic anticancer drugs such as *melphalan* and the evaluation *in-vivo.*

## Introduction

Although chemotherapy is one of the main treatment modalities for cancer, its use is restricted by different problems, such as nonspecificity and multidrug resistance (1). In recent years, nanotechnology-based drug delivery systems have been widely used to improve the treatment outcome of chemotherapy (2, 3). Nanosized delivery systems cause a major change in the pharmaceutical field by improving the therapeutic efficacy and bioavailability of most drugs (4). In this regard, various drug delivery systems, such as liposomal *NPs*, polymeric micelles, dendrimers, carbon nanotubes, aptamers, quantum dots, and polymer compounds have been developed (5). Polymeric and lipidic *NPs* are two US Food and Drug Administration (*FDA*) approved compounds for clinical use and have been successfully used for the encapsulation and release of various drugs in the past two decades (6, 7). The use of amphiphilic polymers for the construction of polymeric *NPs* leads to the formation of *NPs* with a hydrophobic core and a hydrophilic shell. The core-shell-based *NPs* can encapsulate and deliver the poorly water-soluble drugs and increase their blood half-life. Also, they can release the cargoes at a steady rate in the optimal range of drug concentration (8, 9).

Liposomes are spherical lipid vesicles with a bilayer structure of synthetic or natural amphiphilic lipid molecules. They have been widely used in nano-drug delivery systems due to their appropriate features, such as proper safety properties and long blood circulation time, which can be achieved by surface modification using hydrophilic polymers such as polyethylene glycol (*PEG*) (10). *PEG* causes an increase in the molecular weight of conjugates and improves the aqueous solubility as well (11).

Overall, polymeric *NPs* take advantage of high drug loading efficiency, controlled drug release profile, appropriate stability in the blood compartment, and high cellular uptake. However, the biocompatibility and circulation half-life of polymeric *NPs* are two main concerns that must be considered. In contrast, liposomes possess higher biocompatibility and prolonged circulation profile in the blood compartments. Moreover, their surface can be easily modified. Thus, it would be interesting to combine the advantages of liposomes and polymeric *NPs* to develop an advanced hybrid therapeutic system. These systems (*HLPNPs*) can be produced by coating the polymeric *NPs* with lipid layers (12). *HLPNPs* consist of three distinct compartments, including 1) inner hydrophobic core as a drug supplier, 2) interfacial lipid layer as an exceptional biocompatible layer, and 3) outer hydrophilic polymer shell comprising of *PEG* to increase the blood circulation time. These *NPs* have high structural integrity, biocompatibility, and appropriate pharmacokinetic profile which result in an improvement in the anticancer efficacy of chemotherapeutics (13). 

In this study, *melphalan*-loaded *HLPNPs* were synthesized and the drug delivery system was optimized using a central composite design (*CCD*) which is one of the elements of response surface methodology (*RSM*) (14). *Melphalan* is a hydrophobic anticancer drug used for the treatment of ovarian cancer (15, 16). The system (*HLPNPs*) utilized poly lactic-co-glycolic acid (*PLGA*) as the polymer to encapsulate *melphalan*. *PLGA* was used due to its biodegradability and a high potential for loading hydrophobic drugs. *Phosphatidylcholine* was also used as the lipid constituent for coating the polymer core and as a biological membrane to improve the penetration of the *NPs* (17). In addition, polyvinyl alcohol (*PVA*) was used as the surfactant. The effects of various variables (*PLGA*, lipid, and *PVA*) were evaluated on the size and drug entrapment efficiency (*EE %*) and the optimized formulation was characterized in terms of drug release and cytotoxicity effects. This study aimed to investigate the cytotoxic efficacy of the *NPs* loaded with *melphalan*. Also, it was assumed that using *HLPNPs* enhances the therapeutic effects of *melphalan*. 

## Experimental

*Materials*

*Melphalan* was purchased from CELON LAB Co. (India). Soya lecithin, methyl thiazole tetrazolium (*MTT*), and polyvinyl alcohol (*PVA*) were prepared from Sigma-Aldrich Co (Germany). Roswell Park Memorial Institute-1640 (*RPMI-1640*), and Dulbecco’s Modified Eagle’s Medium (*DMEM*) were purchased from Invitrogen (Germany). Human ovarian cancer *A2780CP* and *SKOV3* cells were obtained from the Pasteur Institute of Iran. Moreover, organic solvents such as chloroform, methanol, and isopropanol were purchased from Merck Co (Germany). Furthermore, (*PLGA*) at 50:50 molar ratio was purchased from Iran Polymer and Petrochemical Institute. Deionized water was used to prepare the *NPs*. All of the materials used were of analytical grade.

*Experimental design*

The amount of lipid, polymer, and *PVA* used for the construction of *HLPNPs*, was optimized using an experimental design by evaluating their effects on the physical and chemical properties of the *HLPNPs*. For this purpose, 20 experiments were designed using *RSM* and *CCD* and Design Expert 10.0.7 Trail software. The effects of three factors [Polymer (A), Lipid (B) and *PVA* (C)] as independent variables at three different levels (1, 0, -1), two axial points (-α, +α), and six replicates at the central point were studied to estimate the trial error and calculate repeatability. Physical and chemical properties, such as particle size (Y_1_) and drug *EE%* (Y_2_) were selected as the dependent variables. Table 1 presents the design parameters. The optimum condition was considered when the particle size (Y_1_) was minimum while the drug *EE%* (Y_2_) was the maximum (18).

*Fabrication and preparation of the HLPNPs drug*

*NPs* were prepared using the nanoprecipitation method (13). Briefly, different amounts of lipid (2.3-5.7 mg/mL) in an aqueous solution containing different amounts of *PVA* (0.3-3.7% V/W) were used to prepare the aqueous phase. Also as the organic solvent, different amounts of polymer (3.3-6.7 mg/mL) were dissolved at the constant content of the drug (1 mg/mL) in chloroform. Then, the organic phase was added dropwise (to avoid aggregation) to the aqueous phase under high stirring and ice bath conditions (using a homogenizer at a speed of 12000 rpm). The obtained solution was stirred at ambient temperature for 2 h to make the binding and remove the organic solvent. To ensure that any organic solvent was removed, the obtained nanosuspension was centrifuged three times by Amicon filter 12 kDa. In the next stage, the obtained precipitation was suspended again in deionized water to examine their size and shape. Afterward, the resulting solution was placed on ice and sonicated (5 min, 60 HZ) to obtain more homogeneous *NPs*. 

*HLPNPs Specifications*

Particle size, polydispersity index (*PDI*), and zeta potential were determined using the Zetasizer device (Nano ZS3600, Malvern Instrument Ltd, UK) based on the method described previously (19). The samples were placed in the analysis cell. The experiment was conducted at ambient temperature with a 90° detection angle. Each experiment was repeated three times, and their responses (nanoparticle size) were measured. Finally, the mean value of each measurement and standard deviation (SD) were calculated.

*Nanoparticles morphology*

Transmission electron microscopy (*TEM*) (Hitachi, Japan, H9500 (JAPE)) was used to detect the structure of the prepared *NPs*. For this purpose, 1 mg/mL of the nanoformulation was prepared in PBS, from which a drop was injected into the device, and the morphology of the nanoliposome on the sample was investigated by producing high-energy electron beams.

*Determination of the drug entrapment efficiency *

To evaluate the amount of the encapsulated drug, 2 mL of each formulation (HLPNPs and its blank sample) was centrifuged (Ultra Centrifuged –UCEN, Iran) at 20,000 rpm and 4 °C for 30 min. The supernatant was obtained and the optical absorbance of the supernatant of each formulation was read by spectrophotometer (UV-160 IPC, Shimadzu, Japan) at the wavelength of 260 nm (20). The following equations were used to calculate the encapsulation and loading efficiencies of *melphalan*. (21): 

$\mathrm{Encapsulation efficiency}\left(\%\right)=\frac{\mathrm{The drug encapsulated in a nanoparticle}}{\mathrm{Amount of drug added initially}}\times 100$ Equation 1. 

$\mathrm{Loading efficiency}\left(\%\right)=\frac{\mathrm{Amount of loaded drug in nanoparticles}(\mathrm{mg})}{\mathrm{Weight of nanoparticles}(\mathrm{mg})}\times 100$ Equation 2. 

**Figure 1:**
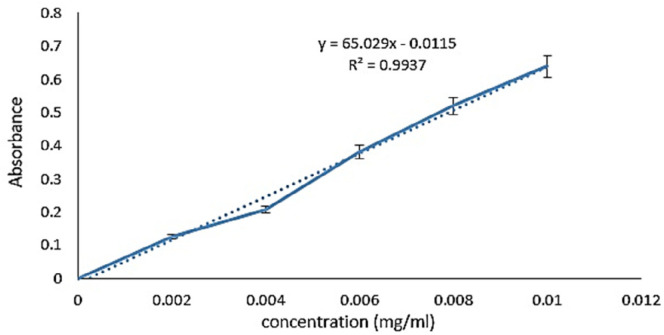
Standard curve of *melphalan*

To obtain the standard curve, different concentrations of *melphalan* were prepared by serial dilution method. The light absorption of the samples was read at 260 nm, and the results were plotted as a standard curve using excel software (Figure 1). The results were obtained from at least three independent experiments.

*Analytical method validation parameters*

In this study, validation parameters of the *melphalan* examination containing range (2-10 µg/mL), linearity, and limit of quantification (*LOQ*) were investigated. Equation 3 was applied to calculate *LOQ* and its value computed for *melphalan* using the information of calibration curve (22):

LOQ=10 σS Equation 3.

Where σ and S are the standard deviation of the response and the slope of the calibration curve respectively.

*Evaluation of melphalan release from the hybrid nanoparticles*

The dynamic diffusion method of the dialysis membrane was used to measure the drug release *in vitro* (23). Two mL of optimal formulation, free drug, and its blank sample were separately poured into three dialysis bags (Mw: cutoff 12 KDa). Then, the dialysis bags were separately immersed into vessels containing 25 mL of phosphate buffer saline (*PBS*, pH 7.4), and stirred for 48 h (200 rpm and 37 °C). At different time intervals, 2 mL of *PBS* was replaced with 2 mL of the fresh buffer. The absorbance of the samples was measured at 260 nm, and the drug release was determined.

*Evaluation of the cytotoxic effect of the formulations*

Cytotoxicity of the *HLPNPs* of *melphalan* and the free drug was evaluated by *MTT* assay on ovarian cancer cell lines (*A2780CP* and *SKOV3*). *A2780CP* and *SKOV3* cells were initially cultured in *RPMI-1640* and *DMEM*, respectively. Then, 100 µL of the suspension containing 10^4^ cells was transferred into 96-wells and incubated at 37 ℃ in a 5% CO_2_ incubator. After 24 h, the culture media was removed and the media containing different concentrations (4.25, 8.5, 17, 34, and 68 µg/mL) of *melphalan*-loaded *HLPNPs*, its control, and the standard drug were added to the wells and incubated for 48 and 72 h. Afterward, the culture medium was discarded, and 100 μL of *MTT* solution (0.5 mg/mL) was added and incubated for 3 h (37 °C, 5% CO_2_). Next, the *MTT* solution was removed, and 100 μL isopropanol was added to dissolve the formazan crystals. The absorbance of the samples was then read at 570 nm by an ELISA reader (Bio Tek. Instrument, USA). The amount of the half-maximal inhibitory concentration (*IC*_50_) was calculated by using the statistical Graph-pad prism program.

Moreover, Equation 4 (24) was used to calculate cell viability. The results were obtained from three independent experiments.

$\mathrm{cell viability}=\frac{\mathrm{mean of absorbance of the treatment group}}{\mathrm{mean of absorbance of the control group}}\times 100$ Equation 4.

*Statistical analysis*

All results are expressed as mean ± standard deviation (SD, n = 3). The data were analyzed and evaluated by ANOVA using Design Expert 7 software, and *p*-value < 0.05 was considered to be statistically significant, with a 95% confidence interval. 

## Result and Discussion

*Mechanism of the single-step nanoprecipitation method for the production of the hybrid nanoparticles*

**Figure 2:**
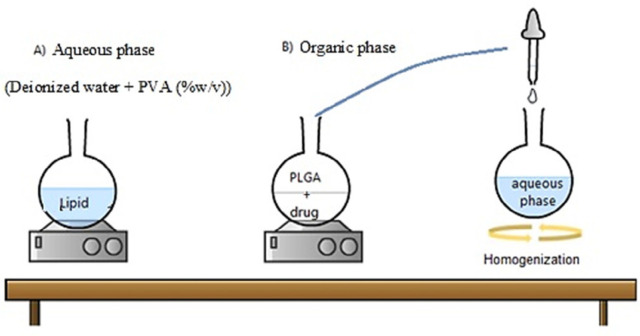
Scheme of the preparation process of the *HLPNPs* using the single-step nanoprecipitation

The nanoprecipitation method is one of the fast and repeatable ones for preparing the *HLPNPs* (Figure 2) (25). This method was mainly based on the polymer precipitation from the lipophilic solution which was a combination of polar solvent and water. In particular, the used polymer (*PLGA*) was precipitated as a hydrophobic core to encapsulate the less water-soluble drug. Notably, adding a lipid layer between the *PLGA* polymer core and *PEG* shell results in i) restriction of the release of small drug molecules from the polymer core which improves the encapsulation and loading efficiencies, and ii) reduction of the water penetration into the polymer core, and as a result, reduction in the hydrolysis rate of the *PLGA* polymer which causes a slow drug release from the *NPS*.

*Validation parameters*

Based on the linear equation taken from the *melphalan* calibration curve the values of σ and S were (0.0346 and 65.02 respectively). Consequently, according to Equation 3, the amount of *LOQ* was obtained at 5.32 µg/mL.

*Analysis and optimization of the central composite design*

Statistical analysis based on *RSM* was used to predict the most appropriate model to describe the response surfaces (nanoparticle size and *EE%*). Each experiment was repeated three times, and the response surface was determined in each experiment. Table 2 presents the outputs. The results of the experimental design indicated that the designed system was affected by the amount of lipid, polymer, and *PVA*, resulting in various drug *EE%* and *NPs* sizes. As shown in Table 3 and Table 4, the best state for each quadratic model response compared to the linear model was the quadratic two-factor model, which had the highest correlation coefficient (R^2^). Thus the quadratic model was selected to describe binary interactions of independent variables on each response.

*Effect on the size of the nanoparticles*

The *NPs* size is imperative to determine the efficiency of loaded therapeutics where *NPs* with a smaller size have a higher chance to internalize into cells, resulting in the higher intracellular concentration of the loaded therapeutics (26). *NPs* with a size smaller than 300 nm are effectively up taken by target cells and exert their pharmaceutical activity (27). 

In the present study, the *NPs* size for the 20 synthesized formulations was in the range of 92.01 – 194.03 nm. According to Table 2, the smallest and largest *NPs* were produced in experiments 20 and 10, respectively. The proposed quadratic model to describe the effect of the independent variables on the particle size is presented in Equation 5:

Size=134.10+13.82 A+16.45 B-21.11 C+2.40 AB-4.23 AC-0.613 BC+2.03A2+1.31B2-1.41C2 Equation 5.

Statistical results demonstrated that by increasing the amount of polymer (A) and lipid (B), the particle size with positive coefficients also increased, as shown in Equation 5.

**Figure 3:**
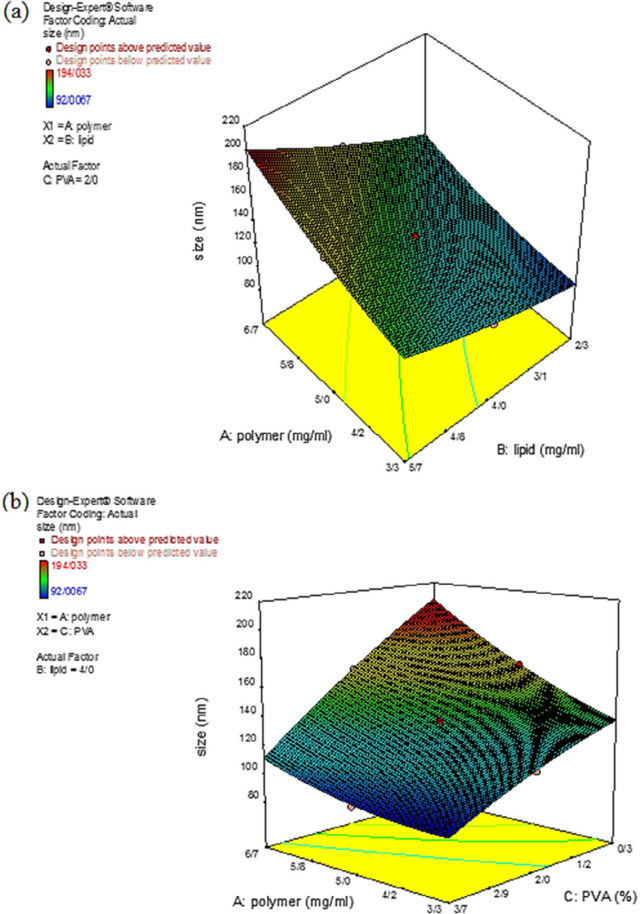
Three-dimensional curve of the effect of the independent variables on the response surface Y_1_. (a) Interaction of polymer and lipid concentration and (b) Interaction of polymer and polyvinyl alcohol concentration

Analysis of variance in the statistical calculations does not express the results with 100% certainty, thus the results are expressed with a percentage of probability. Therefore, by increasing the *F-*value and decreasing the *P-*value, the importance of the *NPs* size would be significant and could be considered as a parameter affecting the process. Accordingly, concerning the results from data variance in Table 5, all three independent variables were found to affect the *NPs* size. Figure.3 depicts the three-dimensional (3D) curve of the particle size response surface for a better understanding of the binary interaction of the independent variables on the response surface. According to Figure.3a, at the high polymer concentrations, the particle size was risen by increasing the lipid concentration. This increase in the particle size might be due to increasing the viscosity of the solution, which in turn caused an increase in the liquid phase resistance of the particle dispersion. Consequently, the particle size can be increased by increasing the interconnection of particles among each other. Therefore, by increasing the number of particles, the interconnection rate between particles was increased, resulting in the production of larger *NPs* (28, 29). Also, by increasing the viscosity, the evaporation rate of the organic solvent was decreased, and particles with larger sizes were produced (30, 31). Figure 3b depicts that by increasing the initial concentration of the surfactant, the particle size decreased. This decrease in the particle size could be explained by the fact that at high concentrations of surfactant, the surfactant molecules tend to be accumulated and were sufficient to coat the *NPs*. Hence, the surfactant activity increased and exert a significant effect on the *NPs* size (32, 33).

*Effect on the drug entrapment efficiency*

Encapsulation efficiency is a critical factor that affects the efficacy of the drug delivery carrier (34). For example, low drug entrapment efficiency in polymeric carriers causes poor treatment outcomes and drug release properties and, as a result, insufficient efficacy of drug delivery systems (19).

In the present study, *EE%* was calculated for all the prepared formulations to evaluate the effects of the independent variables (polymer, lipid, and *PVA*) on the drug *EE%* at different concentrations where the drug concentration was constant. Variation in the *EE%* at different concentrations was described by Equation 6:

EE=88.48+5.00 A-0.11 B-0.55 C+1.97 AB+0.25 AC+0.12 BC-1.07A2+0.17B20.28C2                     Equation 6.

**Figure 4:**
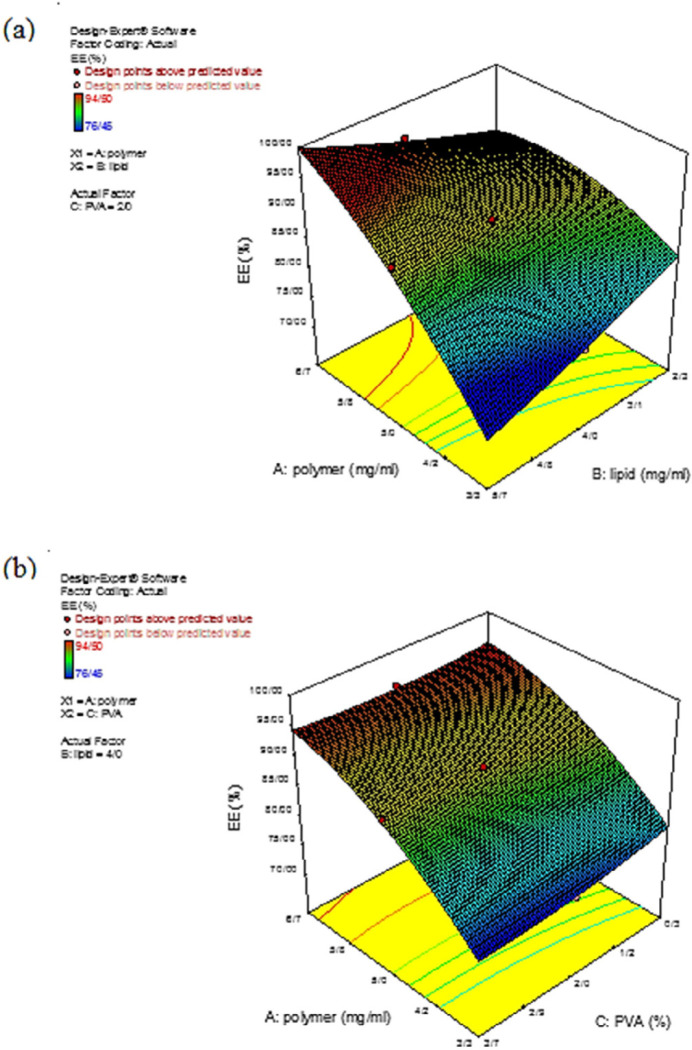
Three-dimensional curve of the effect of the independent variables on the response surface Y_2._ (a) Interaction of polymer and lipid concentration and (b) Interaction of polymer and polyvinyl alcohol concentration

According to Table 2, Experiments number 10 and 14 exhibited the highest and lowest *EE%*, respectively. Regarding the importance of the *F-*value parameter and statistical results reported in Table 6, the two independent variables of polymer concentration (A) and amount of surfactant (C) had significant effects on the *EE%* (*p-*value < 0.05). Figure 4 demonstrates the impacts of the independent variables on the drug *EE%*. As can be seen in Figure 4a, increasing the polymer concentration, the *EE%* also increased. This could be a result of the fact that by increasing the polymer concentration, the encapsulation spaces for the drug also increased; hence, a relatively compressed matrix was created. Also, the hydrophobicity of *melphalan* helped to achieve a high *EE%.* According to the previous research, polymeric core and the drug hydrophobicity, as the two significant factors, promoted the *EE%.* Furthermore, changes in the lipid content had no significant effect on *EE%* and only affected the lipid thickness which resulted in a partial drug release from the nanoparticle (6). As Figure.4b demonstrated that by decreasing the polymer concentration and increasing the surfactant content, the *EE%* decreased. These results from the fact that with increasing the surfactant concentration, the solubility of the drug from the organic phase to the aqueous phase increased, which caused a reduction in the viscosity, and consequently, a reduction in permeation during the process. This issue, in turn, led to a decrease in *EE%* (35).

*Optimization*

**Figure 5:**
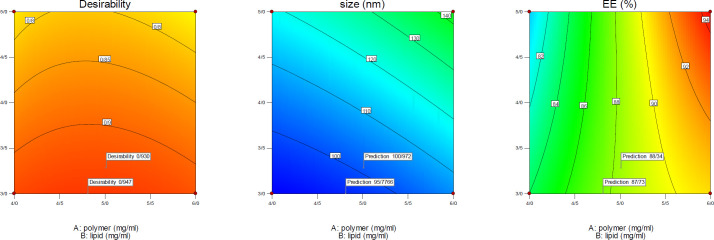
Optimization plots of the response surfaces and desirability of the optimal values

**Figure 6:**
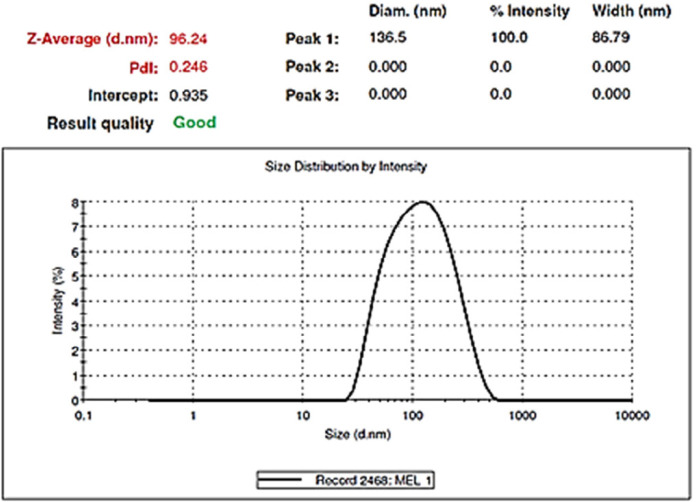
Size distribution of the *HLPNPs* with the values ​​predicted by software

After evaluating the response surfaces by analyzing variance, numerical optimization was performed by applying optimal limitations and specifications for independent variables. Then optimal response surfaces were obtained with the values ​​predicted by the software (Figure 5). Then, the formulation of the *HLPNPs* with the predicted values was prepared, and the *NPs* size, *PDI* (Figure 6), and *EE%* were measured and calculated. Table 7 reports values ​​predicted by the software and actual values ​​of the response surfaces. According to the data presented in Table 7, there was no significant difference between the actual values ​​of the response surfaces and the predicted values. Therefore, the efficiency of encapsulation and loading of *melphalan* was obtained to be 84.43 ± 3.67% and 2.98 ± 1.3%, respectively.

Moreover, the desirability of the optimized values ​​was 0.947. In general, it could be concluded that those *NPs* used as a carrier for anticancer drugs, can easily reach the cell membrane and increase the drug concentration at the cell surface compared to the standard drug. consequently, such a condition increased the therapeutic effect of the anticancer drug. Hence, a little encapsulation efficiency would be of high importance.

*Morphology and zeta potential of the optimal formulation*

**Figure 7:**
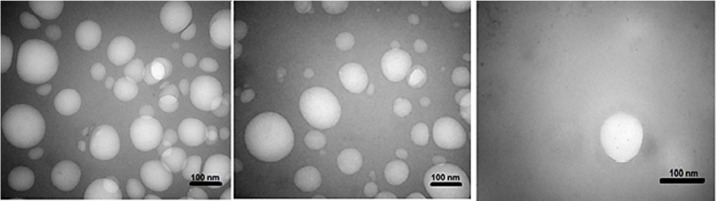
*TEM* micrograph of the *HLPHNPs* containing *melphalan*

**Figure 8:**
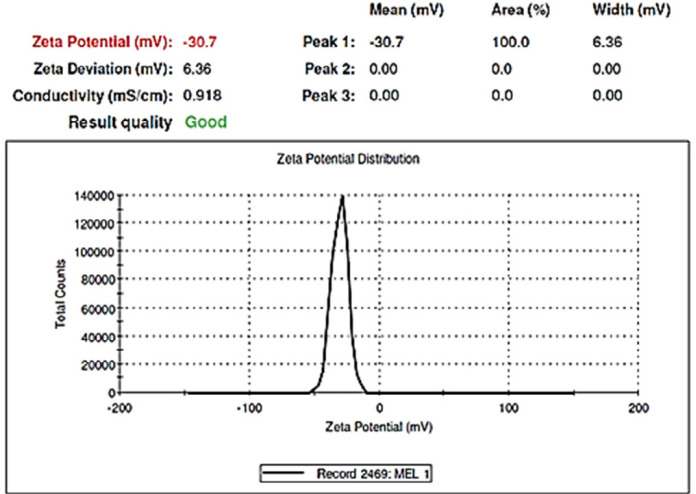
Surface charge potential of the optimal Nano formulation

*TEM* was used to identify the morphology of the optimized hybrid *NPs*. Figure 7 shows images obtained from *TEM*, indicating a small ring of lipid covering around the polymer core. Moreover, it could be seen that the prepared *NPs* had a smooth surface, uniform, and integrated pattern with the spherical structure, suggesting a slow release of the drug. There was a difference, but not significant, between the particle size obtained from *TEM* (94.41 nm) and those obtained by zeta sizer (96.24 nm) (Figure 6). These results were somewhat consistent with the results of similar research, reported previously (36, 37). Zeta potential determines the stability of the colloidal nanosuspensions. Figure 8 depicts the zeta potential of the optimized value attributed to the nonionic nature of *PVA* compared to the anionic nature of phosphatidylcholine. Additionally, negative zeta potential created a large repulsive force between *NPs*, prevented their aggregation, and resulted in their stability (38).

*Drug release study (in-vitro)*

**Figure 9:**
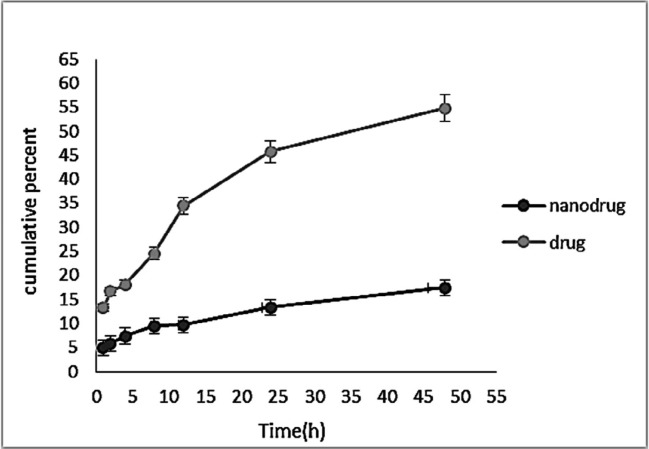
Release profile of *Melphalan* from *HLPNPs* free drug using the dialysis method within 48 h at 37 °C

Controlled-release drug delivery systems have remarkable advantages in comparison with conventional dosage forms. These systems 1) cause a significant decrease in the dosing frequency and provide more convenience for patients, 2) cause a minimum in the fluctuation of drug concentration *in-vivo* and preserve the concentration of drugs within the proper range, 3) can deliver drugs site-specifically, and 4) can reduce the drug side effects (19). Also, drug release from nanocarriers is a critical factor affecting the therapeutic outcome (39). Figure 9 demonstrates a pattern of sustained *melphalan* release (for both the standard and encapsulated forms) at any time point. According to the Figure, the rate of drug release from the *NPs* was much lower than that of the free drug release, indicating that the *NPs* were able to encapsulate the drug, and release it in a controlled manner in that only 17.39% of the encapsulated drug was released after 48 h. The drug release from the *NPs* was initiated with a burst release, in which 29% of the total release occurred in the first hour of the study. This could result from the release of the adsorbed drug to the *NPs* surface. Releasing continued at a reduced rate until the end of the study. Lack of the rapid release of the drug from the *NPs* suggested the proper interaction of *melphalan* with *HLPNPs*. Overall, the pattern of drug release from the *NPs* indicated the potency of the particles as a controlled drug delivery system.

*In-vitro cell viability and IC*_50_ *of nanoformulation*

**Figure 10:**
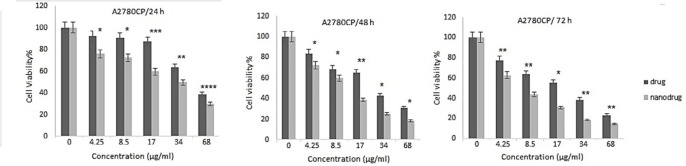
Cytotoxicity effects of free *melphalan* and *HLPNPs* loaded with melphalan on the A2780CP cell line after 24, 48, and 72 h of incubation. Data is expressed as mean ± SD (n = 3). ^*^(*p* < 0.05), ^** ^(*p* < 0.01), and ^*** ^(*p < *0.001) indicate a significant difference with *HLPNPs*

**Figure 11:**
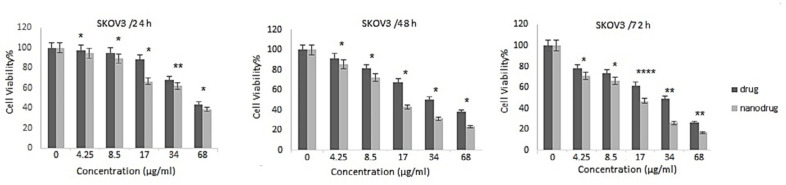
Cytotoxicity effects of free *melphalan* and *HLPNPs* loaded with *melphalan* on the *SKOV3* cell line after 24, 48, and 72 h of incubation. Data is expressed as mean ± SD (n = 3). ^*^(*p* < 0.05), ^**^(*p* < 0.01), and ^***^(*p* < 0.001) indicate a significant difference with *HLPNPs*

**Figure 12:**
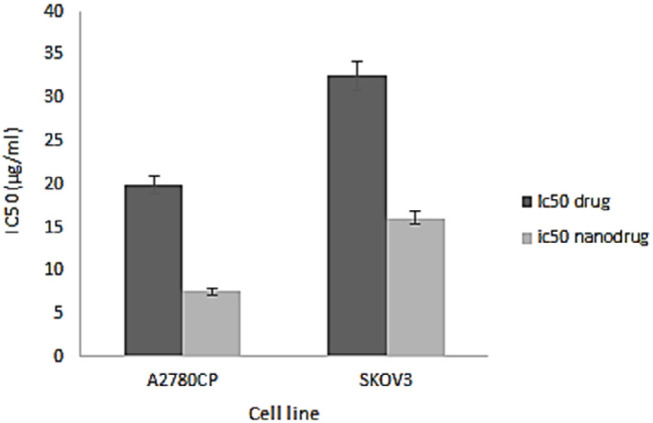
IC_50 _of free *melphalan*, *HLPNPs* loaded with melphalan on the *A2780CP* and *SKOV3* cell line after 72 h. Data is expressed as mean ± SD (n = 3)

*NPs* can increase the therapeutic effects of anticancer drugs because of their capability to enhance the drug concentration in tumor cells. *NPs* perform this by increasing drug circulation time. Moreover, *NPs* can deliver drugs site-specifically *in-vivo, *resulting in the restriction of the drug side effects (26). In the present study, the cytotoxicity effects of *melphalan* and *melphalan*-loaded *HLPNPs* against human ovarian cancer *A2780CP* and *SKOV3* cells were evaluated. As *melphalan* is used for the treatment of ovarian cancer; therefore, *A2780CP* and *SKOV3* cells were used as *in-vitro* models of the disease. The results demonstrated that the cytotoxicity of both formulations (*melphalan* and *melphalan*-loaded *NPs*) was increased in a dose-dependent manner (Figures 10 and 11). Also, the cytotoxicity was found to be cell type-dependent, as both formulations caused higher cytotoxicity in *A2780CP* cells compared to *SKOV3* cells. However, *melphalan*-loaded *HLPNPs* were more potent compared to the standard drug (at the same drug concentration) to inhibit the growth of cancer cells, indicating the potency of the *HLPNPs* to increase the cytotoxicity effects of *melphalan*. Increasing the cytotoxicity effects of the *melphalan*-loaded *NPs* compared to *melphalan*, resulted from the controlled drug release from the *HLPNPs*. Moreover, the nanoformulation at high concentrations inhibited the toxic effects of *melphalan*. This feature increases the maximum tolerable dose, and thus higher concentrations of the drug can be used, which in turn decreases the risk of tumor drug resistance. The cytotoxicity effects were also evaluated by calculating the half-maximal inhibitory concentration (*IC*_50_): the drug concentration required to kill 50% of the cells incubated over the determined period). Figure 12 shows the *IC*_50_ values calculated for both cell lines over the intended time in comparison to the free drug. As seen in the Figure, the *IC*_50_ values of the *HLPNPs* were lower than that of the free drug over time because the free drug quickly passed through the cell membrane while the encapsulated form of the drug chose a specific pathway and released the drug in a controlled way.

**Table 1:** Independent variables and their levels used for central composite design (*CCD*).

**Independent variables Levels**					
	**-1.68**	**-1**	**0**	**1**	**1.68**
A: Polymer (mg.mL)	3.3	4	5	6	6.7
B: Lipid (mg.mL)	2.3	3	4	5	5.7
C: PVA (%)	0.3	1	2	3	3.7
Dependent variables	Constrains				
Y_1_: Particle size (nm)				Minimize	
Y_2_: Entrapment efficiency (%)				Maximize	

**Table 2:** Central composite experimental design matrix and experimental responses. *CCD* methodology represent 20 experiments based on different concentration (mg/mL) of three variables

**Std**	**Run**	**Polymer(mg.mL)**	**lipid(mg.mL)**	**PVA (%)**	**Size(nm)**	**EE (%)**
10	5	1.682	0	0	162.85 ± 5.5	94.5 ± 2.2
4	10	1	1	-1	194.03 ± 2.9	94.45 ± 2.5
8	12	1	1	1	144.07 ± 3.5	93.85 ± 1.6
2	13	1	-1	-1	156.1 ± 5.1	91.5 ± 1.1
6	17	1	-1	1	107 ± 2.4	90.55 ± 1.8
20	1	0	0	0	134.29 ± 2.6	88.45 ± 2.4
11	2	0	-1.68	0	108.69 ± 1.9	88.45 ± 1.3
17	4	0	0	0	136.13 ± 1.1	88.65 ± 1.7
13	7	0	0	-1.68	165.74 ± 5.1	90.01 ± 2.1
16	8	0	0	0	134.29 ± 2.5	87.89 ± 1.4
18	11	0	0	0	133.77 ± 3.3	88.5 ± 1.12
15	16	0	0	0	134.26 ± 2.8	88.4 ± 2.5
12	18	0	1.68	0	164.46 ± 3.1	89.5 ± 1.6
19	19	0	0	0	132.32 ± 3	89 ± 3.1
14	20	0	0	1.68	92.01 ± 2.4	88.58 ± 1.2
3	3	-1	1	-1	154.75 ± 4.4	81.48 ± 2.3
7	6	-1	1	-1	120.19 ± 3.3	80 ± 2.5
1	9	-1	-1	-1	124.88 ± 4.1	86.5 ± 1.2
5	15	-1	-1	1	94.24 ± 2.6	84.45 ± 1.2
9	14	-1.68	0	0	114.36 ± 1.8	76.45 ± 2.3

**Table 3:** The model approved for the response surface Y_1_ (nanoparticle size).

Y_1_( size(nm))						
Source	Std.Dev.	R-Squared	Adjusted R-Squared	Predicted R-Squared	*p*-value		
Linear	4.65	0.9728	0.9677	0.9506	0.0018		
2FI	3.45	0.9879	0.9823	0.9679	0.0067		
Quadratic	1.84	0.9973	0.9949	0.9832	0.0953	Suggested	
Cubic	1.95	0.9982	0.9943	0.7333	0.0239	Aliased	

**Table 4:** The model approved for the response surface Y_2_ (Entrapment efficiency (*EE %*)).

Y_2 _(EE%)						
Source	Std.Dev.	R-Squared	Adjusted R-Squared	Predicted R-Squared	*p*-value	
Linear	1.85	0.8631	0.8375	0.7498	100.16	
2FI	1.34	0.9418	0.9149	0.8722	51.17	
Quadratic	0.62	0.9905	0.982	0.9379	24.87	Suggested
Cubic	0.33	0.9984	0.9948	0.9957	1.72	Aliased

**Table 5:** Results of analysis of variance for the response surface Y_1_ (size of the *NPs*).

Source	Sum of Squares	df	Mean Squares	F Value	*p*-value Prob> F	
Model	12698.91	9	1410.99	414.75	< 0.0001	significant
A-polymer	2607.24	1	2607.24	766.38	< 0.0001	
B-lipid	3694.58	1	3694.58	1086.00	< 0.0001	
C-PVA	6084.58	1	6084.58	1788.53	< 0.0001	
AB	46.04	1	46.04	13.53	0.0043	
AC	143.31	1	143.31	42.12	< 0.0001	
BC	2.86	1	2.86	0.84	0.3811	
A^2^	59.30	1	59.30	17.43	0.0019	
B^2^	24.79	1	24.79	7.29	0.0223	
C^2^	28.71	1	28.71	8.44	0.0157	
Residual	34.02	10	3.40			
Lack of Fit	26.55	5	5.31	3.55	0.0953	not significant
Pure Error	7.47	5	1.49			
Cor Total	12732.93	19				

**Table 6:** Results of analysis of variance for the response surface Y_2 (_*EE%*).

Source	Sum of Squares	df	Mean Squares	F Value	*p*-value Prob>F	
Model	396.60	9	44.07	115.88	< 0.0001	significant
A-polymer	341.34	1	341.34	897.60	< 0.0001	
B-lipid	0.15	1	0.15	0.41	0.5378	
C-PVA	4.10	1	4.10	10.79	0.0082	
AB	30.89	1	30.89	81.23	< 0.0001	
AC	0.49	1	0.49	1.29	0.2828	
BC	0.11	1	0.11	0.28	0.6094	
A^2^	16.50	1	16.50	43.39	< 0.0001	
B^2^	0.40	1	0.40	1.06	0.3270	
C^2^	1.13	1	1.13	2.98	0.1148	
Residual	3.80	10	0.38			
Lack of Fit	3.15	5	0.63	4.81	0.0550	not significant
Pure Error	0.66	5	0.13			
Cor Total	400.40	19				

**Table 7:** Actual and predicted values ​​of the independent and dependent variables for optimal formulation

	Polymer (mg.mL)	Lipid (mg.mL)	PVA (%)	Size (nm)	EE (%)	Desirability
predicted formulation	4.8	3	3	95.77	87.73	0.94
Actual Optimized formulation	4.8	3	3	96.24 ± 2.62	83.43 ± 3.67	……………

## Conclusion

*Melphalan* was loaded onto the *HLPNPs* using a single-step nanoprecipitation method. The *NPs* prepared were evaluated in terms of size, size distribution, zeta potential, *EE%*, and cytotoxicity. Results indicated that the synthesized *NPs* had a high *EE%.* Moreover, the cytotoxicity effects of the loaded drug, compared to the standard drug, increased against the ovarian cancer cells. Overall, the results of this study suggest evaluating the efficacy of the nanoformulation *in-vivo* to confirm the usefulness of the mentioned formulation. 

## Conflict of interest

The authors confirm that this article’s content has no conflict of interest.

## CRediT authorship contribution statement

 Mrs. M mirnezami and Mr. A Heydarinasab: Writing - original draft, Conceptualization and design of the study.

Mr. A Akbarzadeh and Mr. Arjmand: Methodology, Validation, and investigation.

Mrs. M mirnezami and Mr. A Heydarinasab: Analysis of data.

Mr. A Heydarinasab and Mr. A Akbarzadeh: Writing - review and editing, Supervision.
